# Pre-Surgery Depression and Confidence to Manage Problems Predict Recovery Trajectories of Health and Wellbeing in the First Two Years following Colorectal Cancer: Results from the CREW Cohort Study

**DOI:** 10.1371/journal.pone.0155434

**Published:** 2016-05-12

**Authors:** Claire Foster, Joanne Haviland, Jane Winter, Chloe Grimmett, Kim Chivers Seymour, Lynn Batehup, Lynn Calman, Jessica Corner, Amy Din, Deborah Fenlon, Christine M. May, Alison Richardson, Peter W. Smith, Members of the Study Advisory Committee

**Affiliations:** 1 Macmillan Survivorship Research Group, Faculty of Health Sciences, University of Southampton, Southampton, SO17 1BJ, United Kingdom; 2 Faculty of Health Sciences, University of Southampton, Southampton, SO17 1BJ, United Kingdom; 3 University Hospital Southampton NHS Foundation Trust, Southampton, SO16 6YD, United Kingdom; 4 Executive Office, University of Nottingham, Nottingham, NG7 2RD, United Kingdom; 5 Social Statistics and Demography, Social Sciences, University of Southampton, Southampton, SO17 1BJ, United Kingdom; University General Hospital of Heraklion and Laboratory of Tumor Cell Biology, School of Medicine, University of Crete, GREECE

## Abstract

**Purpose:**

This paper identifies predictors of recovery trajectories of quality of life (QoL), health status and personal wellbeing in the two years following colorectal cancer surgery.

**Methods:**

872 adults receiving curative intent surgery during November 2010 to March 2012. Questionnaires at baseline, 3, 9, 15, 24 months post-surgery assessed QoL, health status, wellbeing, confidence to manage illness-related problems (self-efficacy), social support, co-morbidities, socio-demographic, clinical and treatment characteristics. Group-based trajectory analyses identified distinct trajectories and predictors for QoL, health status and wellbeing.

**Results:**

Four recovery trajectories were identified for each outcome. Groups 1 and 2 fared consistently well (scores above/within normal range); 70.5% of participants for QoL, 33.3% health status, 77.6% wellbeing. Group 3 had some problems (24.2% QoL, 59.3% health, 18.2% wellbeing); Group 4 fared consistently poorly (5.3% QoL, 7.4% health, 4.2% wellbeing). Higher pre-surgery depression and lower self-efficacy were significantly associated with poorer trajectories for all three outcomes after adjusting for other important predictors including disease characteristics, stoma, anxiety and social support.

**Conclusions:**

Psychosocial factors including self-efficacy and depression before surgery predict recovery trajectories in QoL, health status and wellbeing following colorectal cancer treatment independent of treatment or disease characteristics. This has significant implications for colorectal cancer management as appropriate support may be improved by early intervention resulting in more positive recovery experiences.

## Introduction

Worldwide 17 million people are living with or beyond cancer. This figure is expected to triple by 2050. If patients are not adequately supported their recovery may be impaired at a cost to patients, those close to them, health and social care [1].

Cancer care is changing to tailored care according to need rather than a single approach for all. There is an increasing emphasis on supported self-management yet little is known about how equipped cancer survivors are for this [2]. Foster and Fenlon developed a conceptual framework of recovery from cancer in which confidence to manage problems and symptoms (self-efficacy: belief in one’s ability to manage illness-related problems) is hypothesised to be central to recovery [3]. A positive association between self-efficacy and quality of life (QoL) has been reported in cross-sectional studies [4–6], however, prospective studies are lacking [7].

While studies have explored QoL following colorectal cancer, most are cross-sectional and few include pre-treatment assessments [8]. Studies typically focus on specific outcomes, such as physical [9] and psychological symptoms [10], comorbidities [9], and presence of a stoma [11] which are associated with poorer QoL. Whilst these studies are important they are limited in describing the impact of cancer treatment on individuals and the process of recovery, or how individuals at risk of poor recovery can be identified or supported.

Previous literature suggests that QoL in the first years following colorectal cancer treatment are similar or better than population norms [7]. However these data report overall means and do not explore distinct groups of patients who may fare better or worse. Studies have begun to examine this by identifying trajectories of recovery for different populations and outcomes. For example, Dunn et al [12] identified 4 trajectories of psychological distress following colorectal cancer with baseline assessment at 5 months; 19% had consistently low levels of distress however the majority (39%) experienced a moderate level of distress which increased rather than decreased over time. Being younger, male, with late stage disease, low education and limited social support was associated with worse distress over time. In a further paper, Dunn and colleagues [13] described trajectories of health-related QoL (HRQoL) and life satisfaction over 5 years among colorectal cancer survivors (no pre-treatment assessment); 4 distinct trajectories were identified for HRQoL (measured by FACT-C), and 19% of respondents reported poor HRQoL throughout the 5 years. Factors associated with poorest recovery included being younger, having low social support, negative cognitive appraisal and low optimism. These findings have begun to enhance our understanding of the nature of longer-term recovery from cancer, however they do not include self-efficacy or assessments before treatment begins.

This paper presents data from CREW, the first large-scale cohort study of a representative group of colorectal cancer patients recruited before surgery and followed up at regular intervals that has a focus on recovery of health and wellbeing. We examine the impact of curative intent treatment on the processes of recovery of health and wellbeing, the role of factors hypothesised to be associated with recovery and implications for how patients can best be supported. The objectives of this paper are to: 1) describe recovery trajectories after colorectal cancer surgery in the first 2 years; and 2) find predictors for these, including self-efficacy.

## Methods

### Study design and participants

A prospective cohort study of colorectal cancer patients recruited from 29 UK hospitals between November 2010 and March 2012 [14, 15]. Eligible patients: a) diagnosis of colorectal cancer (Duke’s A-C, where A = T1/2, B = T3/4 N0 and C = T1-4 N1 or 2, with an R0 resection), b) awaiting initial curative intent surgery, c) ≥ 18 years, d) ability to complete questionnaires. Exclusions: metastatic disease at diagnosis or prior diagnosis of cancer (exceptions: non-melanomatous skin cancer; in situ carcinoma cervix). Written consent obtained and baseline questionnaires were completed prior to surgery wherever possible; follow-up questionnaires were completed at 3, 9, 15 and 24 months post-surgery (longer-term assessments ongoing). Socio-demographic information was collected at consent, including postcode, which was used to calculate the Index of Multiple Deprivation, a measure of neighbourhood deprivation [16]. Clinical and treatment details were collected from medical notes. Ethical approval was granted by the UK NHS Health Research Authority NRES Committee South Central—Oxford B (REC ref: 10/H0605/31).

### Measures

Areas of assessment were informed by our recovery framework [3]. Validated measures were repeated at every time point unless otherwise indicated.

#### Quality of life, health status and personal wellbeing

Quality of Life in Adult Cancer Survivors (QLACS [17]). Part 1 generic domains: negative feelings, positive feelings, cognitive problems, pain, sexual interest/function, energy/fatigue, social avoidance. Summing these domains (reverse-scoring positive feelings) yields Generic Summary Score (GSS). Part 2 (assessed ≥9 months) cancer-specific domains: appearance concerns, financial problems, distress from fear of recurrence, distress from family risk of cancer, benefits of cancer. Summing these domains (excluding benefits of cancer) yields Cancer-specific Summary Score (CSS). Higher scores represent poorer QoL (except positive feelings; benefits of cancer).

The EQ-5D and EQ Visual Analogue Scale (VAS) measure health status [18, 19]. Scores from each of 5 domains are combined and converted into a summary utility index; higher scores indicate better health. A score of 1 for the utility index indicates “full health” (no problems on any of the 5 domains).

The Personal Wellbeing Index–Adult (PWI-A [20]) contains 8 items of satisfaction corresponding to: standard of living, health, achieving in life, relationships, safety, community-connectedness, future security, and spirituality/religion. An overall score of subjective wellbeing is calculated, with higher scores denoting better wellbeing (< 70 represents reduced wellbeing).

#### Psychosocial factors

State-Trait Anxiety Inventory (STAI [21]) consists of 20 items (state only), with a higher total score indicating greater state-anxiety; ≥ 40 has been suggested to indicate clinically significant anxiety [22].

Centre for Epidemiologic Studies Depression Scale (CES-D [23]) consists of 20 items, with a higher total score indicating greater depression; ≥ 20 has been suggested to indicate clinical depression (major and minor) for cancer patients [24].

Positive and Negative Affect Schedule Short Form (PANAS [25]) consists of two 5-item mood scales measuring positive and negative affect. Higher scores represent stronger positive or negative emotions.

Self-Efficacy for Managing Chronic Disease Scale [26] consists of 6 items (S1 Fig). Higher scores indicate greater confidence to manage illness-related problems.

Medical Outcomes Study (MOS [27]) consists of 19 items, 18 of which comprise 4 subscales representing emotional/informational, tangible, affectionate support and positive social interaction. An overall support index is calculated as the mean of all individual items; higher scores denote greater social support.

### Statistical methods

Target sample size was 1000, based on ability to detect a difference of 0.5 of a standard deviation (SD) in the QLACS-GSS (assuming mean 71.2, SD 25.6) [17], with 80% power and 5% significance. This allowed for 30% drop-out and an intra-cluster correlation of 0.05 to take into account cluster effects within sites.

Subscale scores were calculated according to published guidelines where available; otherwise if ≥75% items within a subscale had been completed mean scores were imputed from completed items. Participants with missing questionnaires were included in analyses for time-points for which they provided data; no imputation of missing questionnaires. Many subscale scores were not normally distributed; means and standard deviations are presented, to enable comparison with other studies. The Index of Multiple Deprivation was categorised into quintiles.

Group-based trajectory analyses [28] were used to investigate whether distinct trajectories of outcomes could be identified for QLACS-GSS, EQ-5D utility index and PWI. These are discrete mixture models, which model the outcomes as censored normal data following a polynomial time curve. The optimal number of distinct trajectories for each outcome was determined using the Bayesian information criterion (BIC) [29, 30] to compare model fit (a change in BIC >10 supports the more complex model), while aiming to avoid trajectories containing very few individuals. The shape of each trajectory was assessed to determine whether it was best described by a linear, quadratic or cubic function according to the significance of each term. Statistical significance of model parameters was assessed by the Wald test. Estimated proportions of participants within each trajectory were obtained from the models, with 95% confidence intervals (CI). Date of surgery was taken as time zero and follow-up time calculated using date of questionnaire completion; timing of baseline questionnaire (pre/post-surgery) was adjusted for in all trajectory models.

Multinomial logistic regression was used to determine statistically significant predictors of trajectory group membership from the following domains measured at baseline: (i) pre-existing factors (sociodemographic, clinical, treatment) and (ii) psychosocial factors (anxiety, depression, positive/negative affect, self-efficacy, social support). Factors found to be significant or borderline significant (p<0.1) from univariate analyses were modelled together, and only those which remained statistically significant were retained in the final prediction models. Odds ratios (OR), with 95% CI for each predictor were obtained from the regression models.

## Results

**Fig 1:**
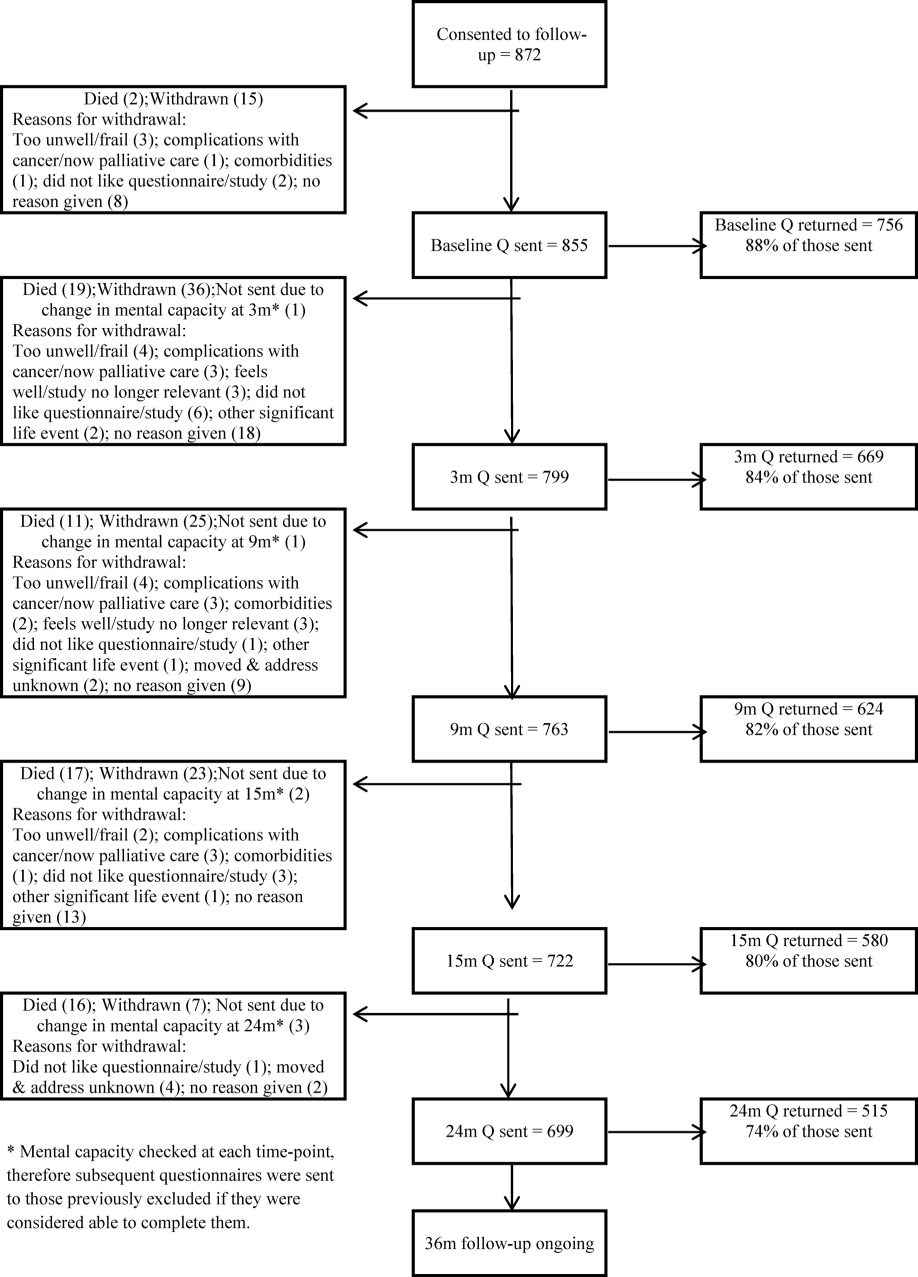
Participant status and questionnaire return rates up to 24 months of follow-up. Number of questionnaires sent and returned at each time-point up to 24 months, with details of deaths and withdrawals throughout the study.

A total of 1,056 participants consented to the study (910 to all aspects of data collection and 146 to collection of medical details only) out of 1,234 invited. Post-surgery, 38/910 were deemed ineligible due to benign or advanced disease, leaving 872 participants for follow-up, of whom 15 withdrew consent prior to baseline data collection (Fig 1). The final denominator for collection of questionnaire data was 857 participants. The sample is representative of eligible patients treated in the recruitment period and includes: 64.6% colon and 35.4% rectal patients (Table 1). 18.4% received neoadjuvant, 34.7% adjuvant therapy and 35.4% had a stoma (most temporary). A total of 39 participants reported having ever used mental health services (5.4% of the 723 who responded to this item on the baseline questionnaire). By 24 months, 79 participants (9.3%) had experienced a recurrence (median 13 months), 65 (7%) had died, and 105 (12%) had withdrawn (Fig 1). Response rates were high at each time point; 809 (94.4%) completed at least one questionnaire from baseline to 2 years and were included in longitudinal analyses. Most participants (592/857, 69%) were consented and completed their baseline questionnaire prior to surgery but due to logistical issues (e.g. those receiving emergency surgery or surgical dates altered without notifying the research nurse) some were enrolled soon after surgery. Overall scores for QLACS, EQ-5D and PWI at each time-point show participants were coping reasonably well up to 2 years post-surgery, with mean scores on QLACS-GSS comparable with other cancer survivors at 15 months [31], with over 40% reporting “full” health on the EQ-5D (compared with 35% reported by Downing et al [32]) at 15 and 24 months, and 65–70% above the cut-off for reduced wellbeing throughout follow-up (Table 2).

**Table 1:** Socio-demographic, clinical and treatment characteristics of CREW participants.

	Number of participants (%)
	N = 857 for socio-demographic & 852 for clinical / treatment details*
**Age (years)**	
≤ 50	55 (6.4)
51–60	118 (13.8)
61–70	311 (36.3)
71–80	265 (30.9)
> 80	103 (12.0)
Unknown	5 (0.6)
*Mean (SD) [range]*	*68*.*2 (10*.*7) [27–95]*
**Gender**	
Male	511 (59.6)
Female	346 (40.4)
**Ethnicity**	
White	662 (77.2)
Non-white	25 (2.9)
Unknown	170 (19.8)
**Domestic status**	
Married / living with partner	534 (62.3)
Single / widowed / divorced / separated	218 (25.4)
Unknown	105 (12.3)
**Employment status**	
Employed	202 (23.6)
Unemployed	34 (4.0)
Retired	513 (59.9)
Unknown	108 (12.6)
**Tumour site**	
Colon	550 (64.6)
Rectal	302 (35.4)
**Dukes stage**	
A	120 (14.1)
B	452 (53.1)
C1	170 (19.9)
C2	99 (11.6)
Unknown–could not be determined ^+^	11 (1.3)
**Stoma**	
No	550 (64.6)
Yes *[Temporary*: *permanent*: *duration unknown]*	302 (35.4) *[182*: *92*: *28]*
**Neo-adjuvant treatment**	
No	690 (81.0)
Yes *[CT only*: *RT only*: *CT & RT]*	157 (18.4) *[19*: *68*: *70]*
Unknown	5 (0.6)
**Adjuvant treatment**	
No	556 (65.3)
Yes *[CT only*: *RT only*: *CT & RT]*	296 (34.7) *[278*: *6*: *12]*
**Biological therapy**	
No	702 (82.4)
Yes (*Cetuximab/Avastin/Panitumumab/Other)*	84 (9.9)
*Unknown*	66 (7.7)
**Number of other long-term conditions ever had** (reported in 3-month questionnaire)	
0	183 (21.5)
1	210 (24.6)
2	148 (17.4)
≥ 3	115 (13.5)
Unknown–not answered on questionnaire	10 (1.2)
Unknown–no 3-month questionnaire	186 (21.8)

CT = chemotherapy; RT = radiotherapy

*Of the original 872 eligible participants who consented to follow-up, 15 withdrew at baseline prior to questionnaire data collection and five patients did not consent to collection of medical details

^+^ Dukes stage could not be determined for 11 participants with small tumours following neo-adjuvant therapy

**Table 2:** Quality of life, health status and personal wellbeing up to 2 years following surgery.

Mean (SD) unless otherwise indicated	Time from surgery^1^				
	Baseline	3 months	9 months	15 months	24 months
	N = 745	N = 548	N = 585	N = 539	N = 491
**Quality of Life in Adult Cancer Survivors (QLACS)**^**2**^					
**Negative feelings**	9.49 (4.20)	9.29 (4.56)	9.25 (4.39)	8.34 (4.08)	8.65 (4.16)
**Positive feelings**	21.18 (5.85)	20.81 (5.89)	21.04 (5.68)	21.35 (6.11)	21.35 (5.82)
**Cognitive problems**	9.14 (4.43)	9.81 (4.78)	9.62 (4.70)	8.84 (4.43)	9.23 (4.37)
**Pain**	9.78 (5.47)	10.16 (5.47)	9.92 (5.62)	8.02 (4.68)	8.29 (4.89)
**Sexual interest / function**	10.65 (5.50)	11.42 (6.00)	11.97 (6.46)	10.54 (6.04)	10.86 (6.12)
**Energy / fatigue**	12.99 (5.52)	13.73 (5.23)	13.07 (5.53)	11.31 (5.11)	11.62 (5.18)
**Social avoidance**	8.30 (4.86)	8.50 (5.12)	8.54 (5.31)	7.34 (4.55)	7.64 (4.65)
**Generic Summary Score**	70.73 (24.60)	73.77 (27.56)	73.00 (28.70)	64.79 (25.84)	66.17 (25.34)
**Appearance concerns**	N/A	N/A	7.06 (4.61)	6.28 (3.78)	6.28 (4.01)
**Financial problems**	N/A	N/A	6.63 (4.47)	6.63 (4.40)	6.35 (3.87)
**Distress–recurrence**	N/A	N/A	11.27 (5.66)	10.12 (5.41)	10.31 (5.22)
**Distress–family**	N/A	N/A	10.87 (6.72)	10.35 (6.44)	10.04 (6.27)
**Benefits of cancer**	N/A	N/A	16.19 (6.46)	16.14 (6.84)	15.95 (6.71)
**Cancer Summary Score**	N/A	N/A	35.75 (16.03)	33.38 (15.02)	32.96 (14.46)
**EuroQoL (EQ-5D)**^**3**^					
**Mobility, n (%)**					
No problems	572 (77.5)	369 (71.1)	N/A	383 (72.3)	336 (71.2)
Some problems	166 (22.5)	148 (28.5)		147 (27.7)	136 (28.8)
Confined to bed	0	2 (0.4)		0	0
**Self-care, n (%)**					
No problems	676 (95.6)	447 (87.5)	N/A	492 (92.7)	444 (94.3)
Some problems	29 (4.1)	63 (12.3)		39 (7.3)	27 (5.7)
Unable to wash	2 (0.3)	1 (0.2)		0	0
**Usual activities, n (%)**					
No problems	495 (67.1)	262 (51.0)	N/A	351 (66.0)	323 (68.3)
Some problems	209 (28.3)	227 (44.2)		174 (32.7)	140 (29.6)
Unable to perform	34 (4.6)	25 (4.9)		7 (1.3)	10 (2.1)
**Pain/discomfort, n (%)**					
No pain	358 (48.7)	247 (47.9)	N/A	346 (65.0)	301 (63.8)
Moderate pain	354 (48.2)	253 (49.0)		178 (33.5)	165 (35.0)
Extreme pain	23 (3.1)	16 (3.1)		8 (1.5)	6 (1.3)
**Anxiety/depression, n (%)**					
Not anxious/depressed	485 (65.8)	345 (67.5)	N/A	394 (73.8)	334 (70.9)
Moderately anxious/depressed	237 (32.2)	158 (30.9)		129 (24.2)	131 (27.8)
Extremely anxious/depressed	15 (2.0)	8 (1.6)		11 (2.1)	6 (1.3)
**“Full health” n (%)**					
No	497 (68.8)	339 (67.7)	N/A	297 (56.8)	273 (58.7)
Yes	225 (31.2)	162 (32.3)		226 (43.2)	192 (41.3)
**Summary index (utility score)**	0.78 (0.22)	0.77 (0.23)	N/A	0.83 (0.20)	0.84 (0.19)
**Overall self-rated health status (VAS)**	70.28 (22.00)	73.91 (17.29)	N/A	79.54 (16.07)	N/A
**Personal Wellbeing Index (PWI)**^**4**^					
**PWI summary score**	78.92 (16.31)	75.15 (19.28)	75.44 (17.44)	75.11 (19.26)	72.93 (20.01)
**PWI reduced wellbeing, n (%)**					
<70 reduced wellbeing	152 (21.3)	165 (31.7)	172 (31.5)	159 (31.9)	156 (35.1)
≥70	563 (78.7)	356 (68.3)	374 (68.5)	340 (68.1)	288 (64.9)

SD = standard deviation; VAS = visual analogue scale; N/A = not available (not all measures collected at every time-point)

^1^ Overall denominators are less than those shown in Fig 1 as data were only included in the cross-sectional analyses presented in the table if the questionnaire was completed within a given time-frame around the due date: pre-surgery or < 3 months post-surgery for the baseline questionnaire, ± 2 months for the 3 and 9-month questionnaires and ± 3 months for the 15 and 24-month questionnaires. Denominators also vary for the different subscales within each time-point. Proportion of participants with missing data on individual subscales ranges from 0.2% (QLACS energy/fatigue) to 10.6% (QLACS sexual interest/function).

^2^ Individual QLACS subscales can range from 4–28; Generic Summary Score can range from 28–196; Cancer Summary Score can range from 16–112. Higher scores for QLACS scales indicate *poorer* QoL, with the exception of positive feelings and benefits of cancer, where higher scores indicate *better* QoL.

^3^ The EQ-5D summary index (utility score) ranges from -0.59 to 1, and the EQ-5D VAS has an overall range of 0–100; higher scores represent *better* health. A score of 1 represents “full health” (no problems on any of the 5 health domains).

^4^ PWI can range from 0–100, with higher scores representing greater wellbeing/satisfaction with life.

### Trajectories of quality of life, health status and personal wellbeing

**Fig 2:**
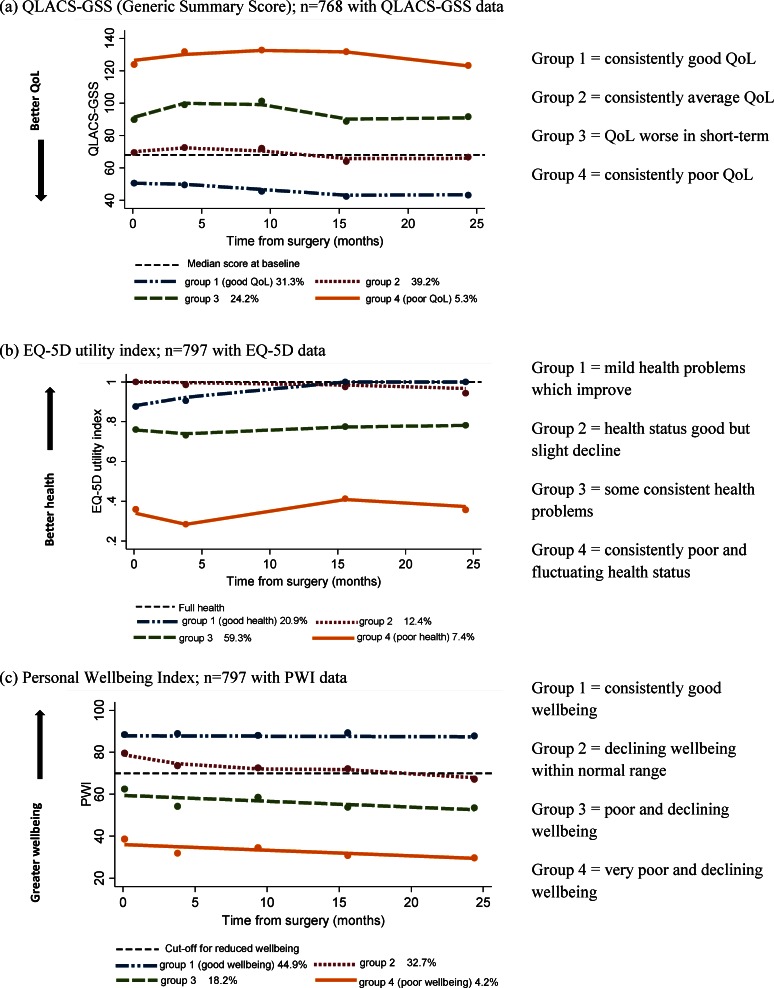
**Estimated trajectories for (a) QLACS-GSS (Generic Summary Score), (b) EQ-5D utility index and (c) Personal Wellbeing Index**.

Four trajectories of recovery from surgery to 2 years (Groups 1–4) were identified for each of the 3 outcome measures representing QoL (QLACS-GSS), health status (EQ-5D) and personal wellbeing (PWI) (S1 Table), and the estimated proportion of the CREW sample in each trajectory was obtained. For QLACS-GSS (similar pattern for all QLACS Part 2 subscales), where low scores indicate better QoL, Group 1 had consistently good QoL (below median baseline score for the whole CREW sample), (31.3%, 95% CI 26.8–35.8%); Group 2 had consistently average QoL (39.2%, 95% CI 34.4–43.9%); Group 3 had worsened QoL in the short-term (≤9 months) which then improved from 15 months, (24.2%, 95% CI 20.1–28.4%); Group 4 had consistently poor QoL, with scores well above the median baseline score (5.3%, 95% CI 3.4–7.3%); (Fig 2A).

For the EQ-5D utility index (Fig 2B), Group 1 began with some (mild) problems but improved by 15 months, reaching a mean score of 1 indicating “full” health (20.9%, 95% CI 16.5–25.3%); Group 2 had consistently good health status which declined slightly from 15 months (12.4%, 95% CI 8.7–16.1%); Group 3 showed consistent health problems over the 2 years (59.3%, 95% CI 54.9–63.6%); and Group 4 displayed fluctuating and the poorest health of the CREW participants (7.4% of CREW sample, 95% CI 4.8–10.0%). For some of the trajectories the change in EQ-5D utility score over time was greater than a published cut-off of 0.08 for a minimally important difference [33].

For the PWI (Fig 2C), Group 1 reported consistently good wellbeing throughout, with scores well above the 70–80 normal range (44.9%, 95% CI 39.1–50.7%); Group 2 had a mean PWI around 80 at baseline which declined to around 70 by 2 years (32.7%, 95% CI 27.5–37.8%); Group 3 showed declining levels of wellbeing which were consistently below the threshold of 70 for reduced wellbeing (18.2%, 95% CI 14.0–22.4%); and Group 4 had the poorest levels of wellbeing consistently <70 and which declined over the 2 years (4.2%, 95% CI 2.3–6.2%).

Across the three outcomes, 56.1% (454/809) were in the best trajectory (Group 1) and 10.7% (87/809) in the poorest trajectory (Group 4) for at least one of the outcome measures.

### Baseline predictors of group membership

Frequencies of sociodemographic, clinical, treatment and psychosocial characteristics according to estimated trajectories for QoL, health status and wellbeing are shown in S2 Table. Lower self-efficacy and a higher level of depression before surgery were statistically significantly associated with poorer trajectories for all three outcome measures adjusting for other significant predictors (Table 3). For example, adjusted OR for QLACS-GSS Group 4 (poorest QoL) versus Group 1 (best QoL) for self-efficacy was 0.32 (95%CI 0.24–0.44, p<0.001), indicating a 68% reduced odds of being in Group 4 versus Group 1 with every unit increase in the score for self-efficacy. Adjusted ORs for self-efficacy for EQ-5D and PWI Group 4 versus Group 1 were 0.60 (0.45–0.81, p = 0.001) and 0.43 (0.31–0.59, p<0.001) respectively, indicating similarly large reductions in odds of being the poorest trajectory with increased self-efficacy. Similarly for depression, adjusted ORs for Group 4 versus Group 1 were 1.20 (1.12–1.28, p<0.001) for QLACS-GSS (indicating a 20% increased odds of being in Group 4 versus Group 1 for every unit increase in the score for depression), 1.13 (1.06–1.20, p<0.001) for EQ-5D and 1.14 (1.07–1.22, p<0.001) for PWI, all indicating a higher risk of being in the poorest trajectories for those with greater depression. The prevalence of clinically significant depression (CESD score ≥ 20) in Group 4 was 77.1% for QLACS-GSS, 66.0% for EQ-5D and 81.5% for PWI, although absolute numbers in this group were small.

**Table 3:** Predictors of trajectory group membership for QLACS-GSS, EQ-5D and PWI: characteristics statistically significant in final multiple regression models for each outcome.

Group 1 totals:	Adjusted odds ratio^1^ (95%CI) relative to trajectory Group 1, p-value^2^								
	QLACS-Generic Summary Score			EQ-5D utility index			PWI		
QLACS-GSS: N = 227	Average QoL (Group 2)	Worse QoL in short-term (Group 3)	Poor QoL (Group 4)	Good health, declining in long-term (Group 2)	Consistent health problems (Group 3)	Poor health (Group 4)	Wellbeing declining within normal range (Group 2)	Poor & declining wellbeing (Group 3)	Very poor & declining wellbeing (Group 4)
EQ-5D: N = 141									
PWI: N = 365	N = 318	N = 183	N = 40	N = 122	N = 480	N = 54	N = 253	N = 147	N = 32
**Age**	N/A	N/A	N/A	N/A	N/A	N/A	0.97	0.97	0.92
Older							(0.95–0.99)	(0.95–1.00)	(0.87–0.98)
							p = 0.017	p = 0.074	p = 0.007
**Gender**	N/A	N/A	N/A	0.58	0.97	0.31	N/A	N/A	N/A
Female vs male				(0.31–1.12)	(0.60–1.56)	(0.11–0.86)			
				p = 0.106	p = 0.902	p = 0.024			
**Deprivation quintile**^**3**^	N/A	N/A	N/A	0.89	1.04	1.48	N/A	N/A	N/A
1^st^ (least deprived), 2^nd^, 3^rd^, 4^th^, 5^th^ (most deprived)				(0.72–1.11)	(0.89–1.22)	(1.05–2.06)			
				p = 0.311	p = 0.617	p = 0.023			
**Live alone**	N/A	N/A	N/A	N/A	N/A	N/A	3.12	3.84	10.55
Yes vs no							(1.76–5.54)	(1.87–7.90)	(2.88–38.68)
							p<0.001	p<0.001	p<0.001
**Number of co-morbidities**^**3**^	N/A	N/A	N/A	0.89	1.53	2.60	N/A	N/A	N/A
0, 1, 2, ≥3				(0.65–1.23)	(1.21–1.93)	(1.62–4.17)			
				p = 0.492	p<0.001	p<0.001			
**Stoma**	2.09	2.88	2.44	0.89	1.85	5.35	N/A	N/A	N/A
Yes vs no	(1.33–3.29)	(1.63–5.07)	(0.88–6.77)	(0.44–1.80)	(1.09–3.13)	(1.98–14.50)			
	p = 0.001	p<0.001	p = 0.087	p = 0.751	p = 0.023	p = 0.001			
**Neo-adjuvant treatment**	N/A	N/A	N/A	N/A	N/A	N/A	1.63	3.39	3.60
Yes vs no							(0.93–2.85)	(1.67–6.89)	(0.95–13.57)
							p = 0.085	p = 0.001	p = 0.059
**Adjuvant treatment**	N/A	N/A	N/A	2.35	1.81	3.16	N/A	N/A	N/A
Yes vs no				(1.25–4.44)	(1.10–3.00)	(1.25–8.00)			
				p = 0.008	p = 0.020	p = 0.015			
**Self-efficacy (Lorig)**^**4**^ **at baseline**	0.60	0.50	0.32	1.17	0.74	0.54	0.60	0.47	0.43
Greater confidence	(0.50–0.72)	(0.41–0.62)	(0.24–0.44)	(0.89–1.53)	(0.61–0.88)	(0.41–0.70)	(0.51–0.71)	(0.39–0.57)	(0.31–0.59)
	p<0.001	p<0.001	p<0.001	p = 0.260	p = 0.001	p<0.001	p<0.001	p<0.001	p<0.001
**Overall social support (MOS)**^**4**^ **at baseline**	0.99	0.98	0.97	N/A	N/A	N/A	0.99	0.97	0.95
More support	(0.98–1.00)	(0.97–1.00)	(0.95–0.99)				(0.98–1.00)	(0.96–0.98)	(0.93–0.97)
	p = 0.096	p = 0.026	p = 0.019				p = 0.046	p<0.001	p<0.001
**Anxiety (STAI)**^**5**^ **at baseline**	1.03	1.05	1.11	0.94	1.00	1.00	N/A	N/A	N/A
Greater anxiety	(1.00–1.06)	(1.02–1.08)	(1.05–1.17)	(0.91–0.98)	(0.98–1.03)	(0.95–1.05)			
	p = 0.022	p = 0.001	p<0.001	p = 0.007	p = 0.832	p = 0.898			
**Depression (CES-D)**^**5**^ **at baseline**	1.07	1.15	1.20	0.99	1.01	1.13	1.03	1.08	1.14
Greater depression	(1.03–1.11)	(1.10–1.20)	(1.12–1.28)	(0.94–1.04)	(0.97–1.04)	(1.06–1.20)	(1.00–1.06)	(1.04–1.12)	(1.07–1.22)
	p<0.001	p<0.001	p<0.001	p = 0.742	p = 0.699	p<0.001	p = 0.032	p<0.001	p<0.001

N/A = not applicable (variable not in final multiple regression model)

^1^ All ORs adjusted for whether baseline questionnaire was completed before or after surgery. ORs for QLACS-GSS also adjusted for stoma, self-efficacy, social support, anxiety and depression. ORs for EQ-5D adjusted for gender, deprivation, co-morbidities, stoma, adjuvant treatment, self-efficacy, anxiety and depression. ORs for PWI also adjusted for age, living alone, neo-adjuvant treatment, self-efficacy, social support and depression.

^2^ p-values from Wald test comparing each trajectory with Group 1

^3^ Deprivation quintile and number of comorbidities fitted as ordinal variables

^4^ Higher scores for self-efficacy and social support indicate *better* levels

^5^ Higher scores for anxiety and depression indicate *worse* levels

Other factors found in the multiple regression to be statistically significantly associated with poorer trajectories for at least one of the outcomes were higher levels of anxiety, and presence of stoma (both QLACS-GSS and EQ-5D), lower social support (QLACS-GSS and PWI), female gender, greater deprivation, more co-morbidities, adjuvant treatment (EQ-5D), younger age, living alone, neo-adjuvant treatment (PWI); (Table 3). There were no statistically significant associations between trajectory group membership and tumour site, Duke’s stage, and positive and negative affect. Recurrence was examined and made only minor differences to the results of the trajectory analyses.

## Discussion

This paper reveals for the first time that pre-surgery level of self-efficacy, i.e. confidence to manage illness-related problems, and depression predict recovery trajectories in all three outcomes of QoL, health status and wellbeing, independent of treatment or disease characteristics. This has significant implications for cancer management as self-efficacy can be enhanced by intervention, and depression may be better supported during the treatment phase to improve the recovery experiences of a significant proportion of colorectal cancer patients. Other important predictors of poor recovery include higher pre-surgery levels of anxiety, lower social support and stoma, which were each significant for two of the outcome domains. Older age was significantly associated with higher levels of wellbeing. This paper identifies who is most in need of intensive support from the point of surgery so resources can be directed accordingly.

Previous research has described the consequences of treatment for colorectal cancer [32] and attention is often focused on supporting recovery once treatment has finished. Self-efficacy has emerged as an independent predictor of recovery of QoL, health status and wellbeing, which suggests health and social care professionals should assess levels of self-efficacy from the point of diagnosis to identify those at risk of poorer recovery. Individuals reporting clinically significant levels of depression also need to receive adequate support to enhance their recovery experiences. The prevalence of depression at cancer diagnosis and its impact on recovery from cancer treatment is not well described. Most patients with depression do not receive potentially effective treatment for their depression [34]. Despite guidance (NICE, 2004 [35], NCCN 2015 [36], NHMRC 2003 [37]) that structured psychological assessment should be undertaken at key points in the individual’s pathway, there is little evidence that this is routinely carried out at diagnosis. Benefit has been demonstrated in managing anxiety and depression using formalised screening [38] and early identification of pre-disposing factors will be key to managing the impact of a cancer diagnosis. Early assessment and intervention to support those experiencing anxiety, depression and low self-efficacy may significantly improve the recovery experiences of those in the worst trajectories. Given that future services will be tailored to meet people’s needs following their treatment it is important that we consider how low self-efficacy and depression will be supported in practice and where barriers to support exist as the longer-term consequences for recovery may be compromised by poor support.

Interventions designed to enhance self-efficacy have been shown to be effective for people living with cancer and treatment-related problems [39]. Dunn and colleagues [12] describe the importance of social support, cancer threat appraisal and optimism in relation to long-term QoL and suggest interventions that target self-efficacy, although they did not measure self-efficacy in their study. Data from CREW provides the first prospective longitudinal data relating to the association between self-efficacy and QoL, health status and wellbeing in colorectal cancer and supports this suggestion. Failing to provide appropriate support to enable people to self-manage the consequences of cancer and its treatment may leave them feeling overburdened, lead to less self-management, greater inequalities, reduced access to services and poorer health and wellbeing [1].

In clinical practice, treatment- and disease-specific factors are frequently used as predictors of outcome, for example; co-morbidities [9, 40–42] having a stoma [43] and symptoms experienced as a consequence of treatment [32]. We found no evidence for independent predictive effects of clinical factors including tumour site and disease stage, nor of personal affect. Assessment of outcomes representing QoL, health status and personal wellbeing in CREW provides a more detailed picture of overall recovery not captured by previous longitudinal studies [12, 32]. This paper demonstrates that it is important to assess psychosocial characteristics including self-efficacy, anxiety, depression and social support, and to do this early in the treatment pathway so that additional support can be offered.

Results of physical symptoms and functioning data that were collected in CREW from 3 months onwards (using EORTC QLQ-C30 [44] and QLQ-CR29 [45] questionnaires) will be presented separately, including the effect of these on overall quality of life. Additional analyses of other clinical outcomes in CREW including recurrence and survival will also be reported separately.

### Study limitations

We set out to recruit all eligible colorectal cancer patients treated with curative intent surgery in 29 cancer centres over a specific period; 91% of all eligible participants were approached. Older patients and the very frail are underrepresented in the sample although there were a number of participants > 80 years. For a number of reasons, some baseline questionnaires were completed after surgery; whilst this maximises the proportion of eligible participants able to be included in CREW, this introduced some variation in baseline responses, and so all analyses were adjusted for time of response to baseline questionnaire. Whilst response rates were high at each time point there has been attrition as would be expected: 10% of the sample has actively withdrawn, 7% have had a recurrence and a similar proportion has died. Incomplete follow-up may have introduced some bias into the longitudinal analyses, although a sensitivity analysis including only the participants with all five completed questionnaires from baseline to 2 years produced similar results for the trajectories. One challenge for research of this type is continuing to engage those who no longer wish to be reminded about their cancer.

### Implications / indications

Novel findings from this study raise important questions for transforming cancer management to identify those at risk of poor recovery before treatment begins and direct services to those most in need. Internationally, cancer care is rapidly changing with the aim of supporting individuals to self-manage. Integral to this are holistic needs assessments, care planning, treatment summaries, cancer care reviews and health and wellbeing events. These assessments and reviews generally focus on physical symptoms, treatment and clinical factors. Where psychological or social domains are examined a problem or need is often quantified, but a person’s capacity to manage is often not considered. The trajectories identified offer a clear opportunity to develop a stepped approach to care offering self-management support to enhance self-efficacy and those identified as having clinical depression, and likely to fare least well, being referred to specialist psychological services. One of the next steps in the research process will be to develop a screening tool based on the risk factors reported here, to identify those individuals at greatest risk of falling into the lower trajectories, irrespective of disease-specific parameters, and use this information to intervene to support them at an early stage in the pathway i.e. soon after diagnosis. This has the potential to revolutionise patient assessment and care-planning and enhance patient care.

## Supporting Information

S1 Fig Self-efficacy for Managing Chronic Disease 6-item Scale (Lorig *et al* 2001). (PDF)

S1 Table Further results from trajectory models: criteria used to determine appropriate number of trajectories for each outcome. (PDF)

S2 Table Frequencies of baseline socio-demographic, clinical, treatment and psycho-social characteristics according to estimated trajectories for QLACS-GSS, EQ-5D and PWI. (PDF)
