# Supplementary material for: Pre-Surgery Depression and Confidence to Manage Problems Predict Recovery Trajectories of Health and Wellbeing in the First Two Years following Colorectal Cancer: Results from the CREW Cohort Study
Source: PLoS One. 2016 May 12;11(5):e0155434. doi: 10.1371/journal.pone.0155434 (PMC4865190; doi:10.1371/journal.pone.0155434)
Supplement: S1 Fig — (PDF) [file pone.0155434.s001.pdf]

S1 Figure: Self-efficacy for Managing Chronic Disease 6-item Scale (Lorig *et al* 2001)

1. How confident are you that you can keep the fatigue caused by your disease from interfering with the things you want to do?	Not at all confident	1	2	3	4	5	6	7	8	9	10 Totally confident
2. How confident are you that you can keep the physical discomfort or pain of your disease from interfering with the things you want to do?	Not at all confident	1	2	3	4	5	6	7	8	9	10 Totally confident
3. How confident are you that you can keep the emotional distress caused by your disease from interfering with the things you want to do?	Not at all confident	1	2	3	4	5	6	7	8	9	10 Totally confident
4. How confident are you that you can keep any other symptoms or health problems you have from interfering with the things you want to do?	Not at all confident	1	2	3	4	5	6	7	8	9	10 Totally confident
5. How confident are you that you can do the different tasks and activities needed to manage your health condition so as to reduce your need to see a doctor?	Not at all confident	1	2	3	4	5	6	7	8	9	10 Totally confident
6. How confident are you that you can do things other than just taking medication to reduce how much your illness affects your everyday life?	Not at all confident	1	2	3	4	5	6	7	8	9	10 Totally confident
