# Supplementary material for: Pre-Surgery Depression and Confidence to Manage Problems Predict Recovery Trajectories of Health and Wellbeing in the First Two Years following Colorectal Cancer: Results from the CREW Cohort Study
Source: PLoS One. 2016 May 12;11(5):e0155434. doi: 10.1371/journal.pone.0155434 (PMC4865190; doi:10.1371/journal.pone.0155434)
Supplement: S1 Table — (PDF) [file pone.0155434.s002.pdf]

S1 Table: Further results from trajectory models: criteria used to determine appropriate number of trajectories for each outcome

Outcome and number of groups	Bayesian Information Criterion (BIC)	Null model for comparison (simpler model with 1 less group)	Change in BIC	Estimated % in smallest group
**QLACS-GSS**				
1	-13230.49	0		
2	-12660.31	1	570.18	35.8%
3	-12459.04	2	201.27	13.1%
**4**	**-12346.46**	**3**	**112.58**	**5.3%**
5	-12326.90	4	19.56	2.4%
6	-12323.12	5	3.78	2.1%
**EQ-5D utility index**				
1	-1108.27	0		
2	-887.93	1	220.34	40.1%
3	-813.95	2	73.98	8.5%
**4**	**-788.31**	**3**	**25.64**	**7.4%**
5	-781.13	4	7.18	3.9%
6	-784.70	5	-3.57	3.2%
**Personal Wellbeing Index**				
1	-12661.28	0		
2	-12143.80	1	517.48	29.3%
3	-11982.36	2	161.44	9.0%
**4**	**-11928.78**	**3**	**53.58**	**4.2%**
5	-11917.15	4	11.63	3.4%
6	-11940.61	5	-23.46	2.9%

Final selected models are highlighted in bold
