# Supplementary material for: Pre-Surgery Depression and Confidence to Manage Problems Predict Recovery Trajectories of Health and Wellbeing in the First Two Years following Colorectal Cancer: Results from the CREW Cohort Study
Source: PLoS One. 2016 May 12;11(5):e0155434. doi: 10.1371/journal.pone.0155434 (PMC4865190; doi:10.1371/journal.pone.0155434)
Supplement: S2 Table — (PDF) [file pone.0155434.s003.pdf]

S2 Table: Frequencies of baseline socio-demographic, clinical, treatment and psycho-social characteristics according to estimated trajectories for QLACS-GSS, EQ-5D and PWI

%s (unless otherwise indicated)	QLACS-Generic Summary Score trajectories				EQ-5D utility index trajectories				PWI trajectories			
	**Good QoL (Group 1) N=227**	**Average QoL (Group 2) N=318**	**Worse QoL in short-term (Group 3) N=183**	**Poor QoL (Group 4) N=40**	**Good health (Group 1) N=141**	**Good health, declining in long-term (Group 2) N=122**	**Consistent health problems (Group 3) N=480**	**Poor health (Group 4) N=54**	**Good well-being (Group 1) N=365**	**Wellbeing declining within normal range (Group 2) N=253**	**Poor & declining wellbeing (Group 3) N=147**	**Very poor & declining well-being (Group 4) N=32**
**Age**												
Mean (SD)	67.7 (8.7)	69.1 (10.1)	66.5 (11.1)	65.2 (12.1)	66.8 (9.1)	68.1 (9.6)	68.7 (10.4)	68.3 (13.0)	69.2 (9.6)	67.5 (10.3)	67.5 (11.1)	64.0 (11.5)
**Gender**												
Male	67.0	62.0	53.5	62.5	63.1	70.5	55.4	63.0	64.4	58.9	49.0	59.0
Female	33.0	38.0	46.5	37.5	36.9	29.5	44.6	37.0	35.6	41.1	51.0	41.0
**Deprivation quintile**												
1st (least deprived)	26.1	20.4	14.4	12.5	22.4	30.3	18.8	7.7	23.9	20.6	14.5	3.1
2nd	22.5	22.0	18.2	12.5	25.4	18.8	21.6	7.7	22.3	17.7	20.7	25.0
3rd	18.9	19.1	21.0	20.0	15.7	20.5	20.3	21.1	19.4	22.6	15.9	15.6
4th	15.8	19.4	19.9	22.5	20.9	12.3	19.2	23.1	17.8	16.1	24.1	25.0
5th (most deprived)	16.7	19.1	26.5	32.5	15.7	18.0	20.1	40.4	16.6	23.0	24.8	31.3
**Live alone**												
No	83.1	78.0	77.1	75.9	78.9	77.2	78.3	77.5	88.4	74.1	67.5	50.0
Yes	16.9	22.0	22.9	24.1	21.1	22.8	21.7	22.5	11.6	25.9	32.5	50.0
**Number of co-morbidities**												
0	34.9	28.4	19.2	10.0	38.3	44.1	22.9	2.4	33.1	23.0	22.3	20.0
1	35.9	30.7	32.7	30.0	34.4	35.3	29.9	35.7	31.0	34.6	28.9	40.0
2	19.0	24.5	23.1	26.7	16.4	15.7	26.3	23.8	20.9	24.9	24.0	24.0
≥ 3	10.3	16.3	25.0	33.3	10.9	4.9	20.8	38.1	15.0	17.5	24.8	16.0
**Tumour site**												
Colon	70.9	61.2	63.7	56.4	67.4	66.4	63.9	51.8	64.6	67.5	60.3	46.9
Rectal	29.1	38.8	36.3	43.6	32.6	33.6	36.1	48.2	35.4	32.5	39.7	53.1
**Dukes stage**												
A	18.2	13.7	13.3	10.3	15.9	13.1	15.3	7.6	17.3	13.2	9.7	22.6
B	55.6	55.3	52.2	56.4	55.8	62.3	52.4	52.8	55.9	50.0	58.6	54.8
C1	16.0	21.7	21.1	15.4	21.0	15.6	20.6	20.7	17.6	23.6	20.7	12.9
C2	10.2	9.3	13.3	17.9	7.2	9.0	11.7	18.9	9.2	13.2	11.0	9.7
**Stoma**												
No	73.8	62.4	55.6	56.4	70.7	72.3	62.1	40.7	67.4	64.5	55.9	50.0
Yes	26.2	37.6	44.4	43.6	29.3	27.7	37.9	59.3	32.6	35.5	44.1	50.0
**Neo-adjuvant treatment**												
No	83.6	83.2	76.8	74.4	86.5	83.5	79.7	70.4	82.0	82.4	76.0	75.0
Yes	16.4	16.8	23.2	25.6	13.5	16.5	20.3	29.6	18.0	17.6	24.0	25.0
**Adjuvant treatment**												
No	66.5	68.1	60.4	59.0	73.0	64.7	64.1	63.0	69.2	63.5	61.0	59.4
Yes	33.5	31.9	39.6	41.0	27.0	35.3	35.9	37.0	30.8	36.5	39.0	40.6
**Self-efficacy[1] (Lorig) at baseline**	8.8 (1.1)	7.5 (1.6)	6.4 (1.7)	4.3 (1.9)	8.2 (1.4)	8.7 (1.1)	7.1 (1.8)	5.3 (2.1)	8.4 (1.4)	7.2 (1.7)	6.0 (1.9)	5.2 (1.8)
**Overall social support[1] (MOS) at baseline**	88.0 (17.6)	81.2 (19.8)	75.5 (22.3)	64.8 (21.8)	83.9 (20.7)	84.7 (17.5)	79.9 (20.7)	75.1 (25.2)	87.8 (18.3)	80.3 (17.2)	70.5 (19.9)	51.1 (28.0)
**Anxiety[2] (STAI) at baseline**												
**< 40**	85.2	63.3	36.3	5.7	71.0	86.2	54.9	25.5	74.6	57.1	35.4	29.6
**≥ 40 (case)**	14.8	36.7	63.7	94.3	29.0	13.8	45.1	74.5	25.4	42.9	64.6	70.4
**Depression[2] (CES-D) at baseline**												
**< 20**	97.2	87.6	54.1	22.9	87.9	94.4	76.7	34.0	90.5	80.3	55.8	18.5
**≥ 20 (case)**	2.8	12.4	45.9	77.1	12.1	5.6	23.3	66.0	9.5	19.7	44.2	81.5

[1] Higher scores for self-efficacy and social support indicate *better* levels; **[2]** Cut-off for STAI $\geq$ 40 indicates clinically significant anxiety and $\geq$ 20 for CES-D indicates clinical depression (major and minor)
